# The Role of Pathogenesis Associated with the Tumor Microclimate in the Differential Diagnosis of Uterine Myocytic Tumors

**DOI:** 10.3390/jcm12124161

**Published:** 2023-06-20

**Authors:** Madalina Bosoteanu, Mariana Deacu, Mariana Aschie, Sorin Vamesu, Georgeta Camelia Cozaru, Anca Florentina Mitroi, Raluca Ioana Voda, Cristian Ionut Orasanu, Sabina Elena Vlad, Roxana Cleopatra Penciu, Sergiu Ioachim Chirila

**Affiliations:** 1 Clinical Service of Pathology, Department of Pathology, “Sf. Apostol Andrei” Emergency County Hospital, 900591 Constanta, Romania; mbosoteanu@yahoo.com (M.B.); deacu_mariana@yahoo.com (M.D.); aschiemariana@yahoo.com (M.A.); sorinvamesu@yahoo.com (S.V.); critian.ionut@gmail.com (C.I.O.); 2 Department of Pathology, Faculty of Medicine, “Ovidius” University of Constanţa, 900527 Constanta, Romania; 3 Academy of Medical Sciences of Romania, 030171 Bucharest, Romania; 4 Center for Research and Development of the Morphological and Genetic Studies of Malignant Pathology-CEDMOG, “Ovidius” University of Constanţa, 900591 Constanta, Romania; drcozaru@yahoo.com (G.C.C.); ank_mitroi@yahoo.com (A.F.M.); sabina.vlad@365.univ-ovidius.ro (S.E.V.); 5 Clinical Service of Pathology, Department of Genetics, “Sf. Apostol Andrei” Emergency County Hospital, 900591 Constanta, Romania; 6 Department of Obstetrics and Gynecology, Faculty of Medicine, “Ovidius” University of Constanţa, 900527 Constanta, Romania; roxanapenciu@yahoo.com; 7 Department of Medical Informatics and Biostatistics, Faculty of Medicine, Ovidius University, 900527 Constanta, Romania; sergiu.chirila@univ-ovidius.ro

**Keywords:** endoglin, FISH, lymphocytes, pathogenesis, uterine tumors

## Abstract

Myocytic tumors of the uterus present vast morphological heterogeneity, which makes differential diagnosis between the different entities necessary. This study aims to enrich the existing data and highlight new potential therapeutic targets regarding aspects related to the pathogenic process and the tumor microenvironment in order to improve the quality of life of women. We performed a 5-year retrospective study, including particular cases of uterine myocyte tumors. Immunohistochemical analyses of pathogenic pathways (p53, RB1, and PTEN) and tumor microclimate using markers (CD8, PD-L1, and CD105), as well as genetic testing of the PTEN gene, were performed. The data were statistically analyzed using the appropriate parameters. In cases of atypical leiomyoma, a significant association was observed between PTEN deletion and an increased number of PD-L1+ T lymphocytes. For malignant lesions and STUMP, PTEN deletion was associated with the advanced disease stage. Advanced cases were also associated with an increased mean CD8+ T cell count. An increased number of lymphocytes was associated with an increased percentage of RB1+ nuclei. The study corroborated clinical and histogenetic data, highlighting the importance of the differential diagnosis of these tumors to improve the management of patients and increase their quality of life.

## 1. Introduction

Among the uterine mesenchymal tumors, the most frequent category is represented by smooth muscle tumors [1]. They include three main entities: leiomyoma (benign tumors originating in smooth muscle cells), leiomyosarcoma, and smooth muscle tumors of uncertain malignant potential (STUMP). The criteria for the first two mentioned representatives are clear, which allows them to be easily classified into one of the categories. However, STUMP is characterized by diagnostic criteria from both previous categories, but without fulfilling them completely [2]. Leiomyomas, in turn, present several types of entity that vary from typical leiomyoma to the form with bizarre nuclei, active mitotic leiomyoma, or metastatic forms, or those that develop intravascularly [1].

The tumor microclimate is a specialized environment, formed of the components of the host and structured by the tumor cells to maintain development and make tumor metastasis possible [3]. Its role is essential in the differentiation of neoplasia, and its epigenetics and dissemination, but also for the evasion of the immune system [4]. All the components of this environment are located in an extracellular matrix containing cytokines, enzymes, growth factors, proteoglycans, and glycoproteins [5]. The dynamic interaction between neoplastic cells and the cellular or acellular components of the tumor microclimate has a major role in the emergence of heterogeneity, clonal progression, and the acquisition of resistance to multiple drugs used in treatment [3].

The aim of the current study is to analyze and compare the main pathogenic pathways, but also the characteristics of the tumor microclimate, with the role of evaluating aspects related to aggressiveness in the context of their differential diagnosis. This evaluation is required due to the different treatment procedures for these tumors, which have many similar elements (nuclear pleomorphism, mitotic activity, imaging, and macroscopic aspects). The motivation for this study is given by the need to describe, as widely as possible, the epidemiological and clinical data of uterine smooth muscle tumors, especially the forms that lend themselves to differential diagnoses between them. The goal is to identify significant correlations for improving the public health of women, by administering targeted treatments and increasing life quality.

## 2. Materials and Methods

We performed a retrospective study over a 5-year period that included particular cases of uterine smooth muscle tumors. These cases were diagnosed in the obstetrics and gynecology departments of our hospital, County Emergency Hospital “Sfantul Apostol Andrei” Constanta. The inclusion criteria were represented by fully documented cases.

The exclusion criteria were represented by cases of necroptic diagnosis or typical leiomyomas, or without evidence of mitotic activity or nuclear pleomorphism.

The information related to clinical and paraclinical data, treatment, and evolution were extracted from the observation sheets of the patients and from the digital database of the hospital.

After performing the surgical interventions, the hysterectomy results were sent for pathological evaluation within the Clinical Pathological Anatomy Service, Constanta. First was a macroscopic description, taking into account the type of surgery, the maximum diameter of the lesions, and the presence of areas of hemorrhage and necrosis. Specimens were processed by embedding them in paraffin and making stained slides with hematoxylin–eosin. The final diagnosis was established according to the WHO criteria corresponding to the year in which the patients were diagnosed. The cases were re-evaluated by two different pathologists, according to the criteria of the latest WHO classification (2020).

The immunohistochemical evaluation was performed at the Center for Research and Development of the Morphological and Genetic Studies of Malignant Pathology (CEDMOG) using the antibodies p53 (SP5 clone, dilution 1:50, HIER-DAB method), RB1 (1F8 clone, dilution 1:50, HIER-DAB method), PTEN (6H2.1 clone, dilution 1:100, HIER-DAB method), CD8 (SP16 clone, dilution 1:50, HIER-DAB method), and PD-L1 (CAL10 clone, dilution 1:50, HIER-DAB method) from Master Diagnostica (Sevilla, Spain), and CD105 (3A9 clone, 1:600 dilution, HIER-DAB method) from Novus Bio (Centannial, CO, USA). The tests were performed on paraffin sections with a thickness of 4 microns, according to the manufacturer’s protocols. Also, FISH analysis of the *PTEN* gene was performed, and the results correlated with those obtained in the immunohistochemical examination using the CEN10 probe from ZytoVision (Bremerhaven, Germany). Interpretation of the results was carried out according to Table 1.

The slides were scanned using a TissueScope LE120 (Huron Digital Pathology, St. Jacobs, ON, Canada) device at ×40 magnification, resulting in whole slide images. These images were visualized using HuronViewer software, version 1.3.1 (Huron Digital Pathology, St. Jacobs, ON, Canada), and 1 region of interest (with the most abundant PD-L1 + lymphocytes) of 1 mm^2^ was selected through the built-in annotation tool. Based on these images, we used IC (immune cell score) quantification to determine the area occupied by PD-L1-positive lymphocytes across the entire tumoral and peritumoral area.

In FISH analysis, two orange signals (CEN10) and two green signals (probes) are normally observed; in cells with gene deletion, there is a decrease in the number of green signals.

Statistical data analysis was performed in SPSS Statistics Version 26 (IBM Corporation, Armonk, NY, USA). Central tendency and variability indicators were used. For categorical variables, univariate data analysis was performed via Fisher’s exact test with a 5 min limit, to reduce the risk of errors, as recommended by the guide developed by IBM SPSS Statistics Exact Tests [6]. For continuous variables, a Mann–Whitney U test and a Kruskal–Wallis H test were performed, as appropriate. To appreciate the association of data, we used the Pearson Correlation Coefficient. Survival estimates were made for a period of 5 years and were calculated using the Kaplan–Meier test. A *p*-value below 0.05 is considered statistically significant.

All patients signed their informed consent at the time of hospitalization. Also, this study was approved by the Ethics Committee of the hospital.

## 3. Results

**Figure 1:**
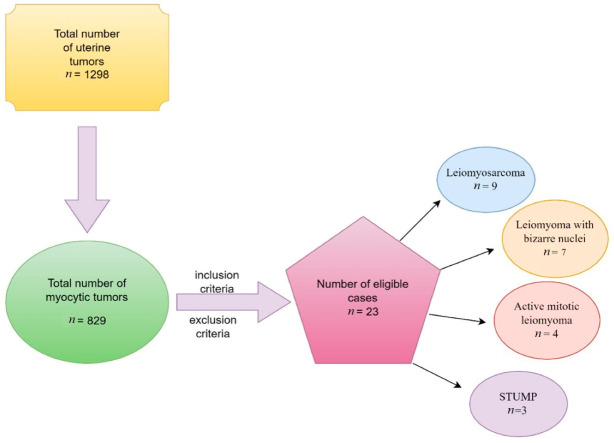
Flow chart of the selected cases: leiomyosarcoma (39.13%), symplastic leiomyoma (30.43%), mitotically active leiomyoma (17.39%), and STUMP (13.04%).

We identified 1298 cases of uterine pathology, of which 829 were myocytic tumors. After applying the inclusion and exclusion criteria, 23 eligible cases were obtained, and they are presented in Figure 1.

The cases of malignant tumors were observed at an older age (sixth decade), unlike the other cases (fifth decade) (*p* = 0.007). The majority of the cases included were women in the fertile period (60.87%). Hormonal status represents a parameter that presented statistically significant correlations with the diagnosis, thus showing that leiomyosarcomas, followed by symplastic leiomyomas, were associated with menopause (*p* = 0.016). A total of 60.87% of the patients had had previous pregnancies, and the rest were nulliparous. The three most common clinical aspects were metrorrhagia (65.22%), pelvic pain (60.87%), and hypermenorrhea (8.70%). Of these, a statistically significant correlation was observed between the presence of hypermenorrhea and the diagnosis of STUMP (*p* = 0.012). Also, an early onset of symptoms was observed (which determined faster presentation to the doctor) in the case of leiomyosarcomas compared to the other tumor entities (*p* = 0.001). The most common comorbidities encountered were arterial hypertension (43.48%), dyslipidemia (43.48%), diabetes mellitus (13.04%), and non-euthyroidism (13.04%) (Table 2).

In the study group, it was observed that older age was associated with the following comorbidities: arterial hypertension (*p* = 0.003), type 2 diabetes (*p* = 0.009), non-euthyroidism (*p* = 0.013), and dyslipidemia (*p* = 0.003). Acute onset, with presentation in the first week to the doctor, was associated with an increased mortality rate (*p* = 0.006).

Sonographically, in the case of leiomyomas, well-defined hypoechoic lesions were observed, located in the continuation of the myometrium. For malignant tumors, hetero-echogenicity was evident, which is why computed tomography (CT) was performed. This investigation revealed an increase in the volume of the uterus due to the presence of imprecisely defined lesions, with areas of hemorrhage and necrosis of varying extents, with low attenuation.

From a surgical point of view, the majority of patients were treated with total hysterectomy (73.91%) (Table 3). In addition to surgical treatment, a third of the patients with leiomyosarcoma (13.04% of cases) also benefited from oncological chemotherapeutic treatment. At the end of this study, 73.91% of patients survived. Death occurred only in the category of patients with malignant tumors (*p* = 0.008). The average survival in these cases was 25.67 months, with the relative risk of these cases by comparison being 5.66 (*p* < 0.001, CI-95: 2.02–15.82).

Most of the lesions (69.57%) were intramural (Table 3). A statistically significant difference was observed in the surgical approach technique to the location of the lesions (*p* = 0.025). Surgeons opted for a total hysterectomy with or without bilateral adnexectomy in cases of intramural localization, and myomectomy in cases of subserosal localization. Also, an association between diagnosis and location was observed, consisting of a preponderance at the intramural level of malignant lesions and those with uncertain malignant potential (*p* = 0.014). The patients who presented with an intramural location of the tumor had faster presentation to the doctor (less than a week) than those with presentation in other locations (*p* = 0.039). In the category of nulliparous patients, an association with intramural localization was observed (*p* = 0.035). The lesions had maximum diameters between 1.5 cm and 28 cm, with an average of 8.48 cm.

**Figure 2:**
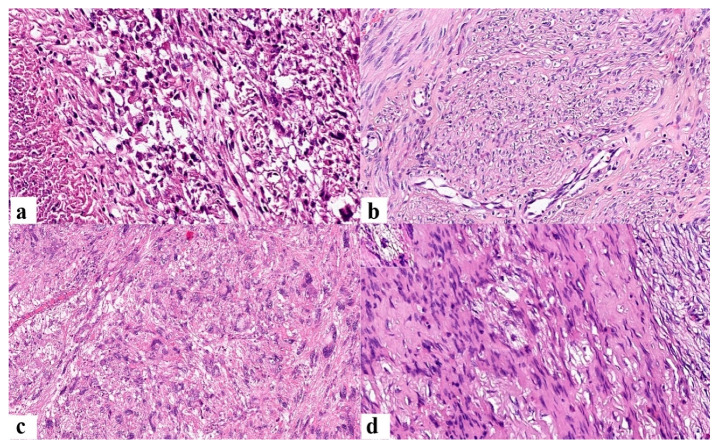
Examination of hematoxylin–eosin staining: (**a**) leiomyosarcoma showing proliferation with high cellularity and atypical mitoses, which is associated with an area of tumor necrosis (Ob. 200×); (**b**) STUMP—dense cellular proliferation with cytoarchitectural atypia and atypical mitoses, without areas of hemorrhage and necrosis (Ob. 200×); (**c**) leiomyoma with bizarre nuclei—on the background of a typical leiomyoma, bizarre nuclei with large sizes are evident, in the absence of atypical mitoses, hemorrhage, and necrosis (Ob. 200×); (**d**) mitotically active leiomyoma—the appearance of typical leiomyoma is associated with the presence of mitoses, without presenting other particularities (Ob. 200×; in the box—typical mitosis, Ob. 630×).

Histopathological examination established the final diagnosis, highlighting, at the same time, the biological potential of the lesions (Figure 2).

**Figure 3:**
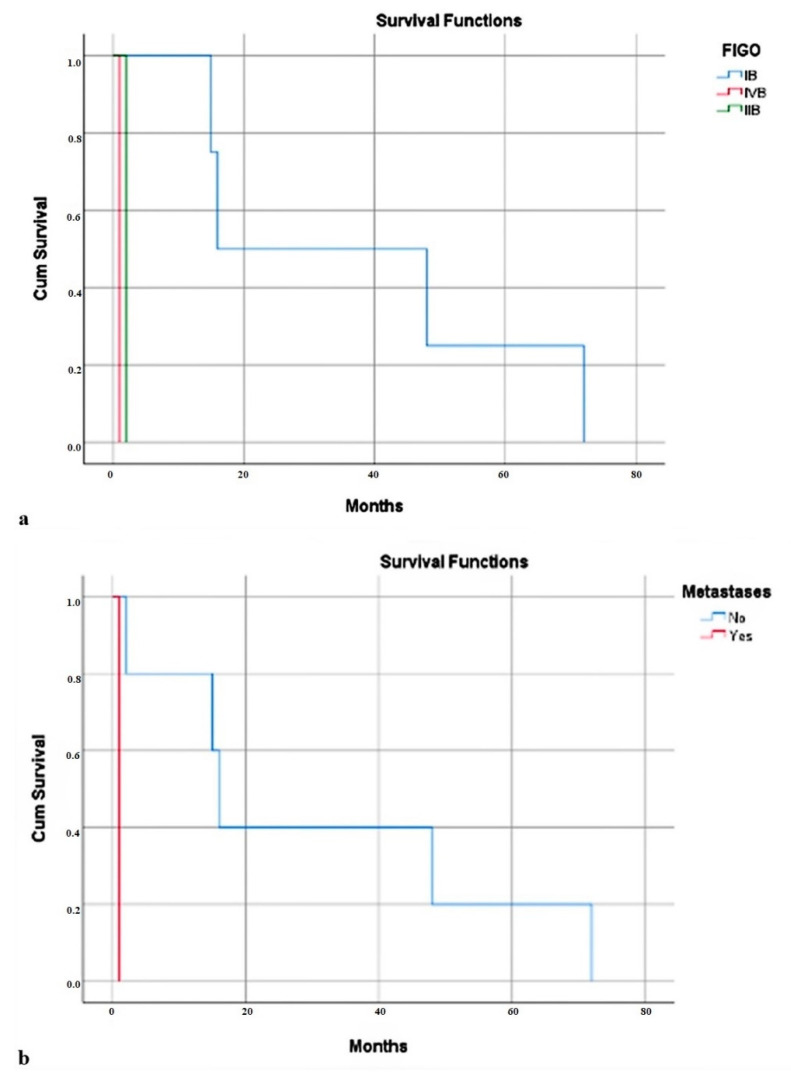
(**a**) Months of survival depending on the FIGO stage. (**b**) Survival depends on the presence of metastasis.

In the case of leiomyosarcomas, most cases had FIGO IB. Increased survival of patients with FIGO IB (37.75 months) was observed, compared to stages IIB and IVB (2 months and 1 month, respectively) (*p* = 0.028) (Figure 3a). One-third of the cases presented lymphovascular invasion. However, 77.78% of cases presented metastases. Their presence was associated with low average of survival (30.60 months vs. 1 month) (*p* = 0.025) (Figure 3b).

The mitotic activity, quantified as the number of atypical mitoses observed in 10 HPF fields in the hotspot, showed an average of 9.91 mitoses (1–35), with a statistically significant difference observed with the diagnosis (*p* = 0.002).

The presence of necrosis, nuclear atypia, and hemorrhage was observed only in cases of malignant tumors and those with uncertain biological potential. An advanced age at diagnosis was associated with the presence of tumor necrosis and nuclear atypia, and with the presence of hemorrhage (*p* = 0.005, *p* = 0.003, respectively *p* = 0.045). The presence of necrosis in these cases was associated with intramural localization (*p* = 0.035) and with the rapid onset of symptoms causing the patients to see the doctor in less than a week (*p* = 0.008). Also, the presence of necrosis was associated with increased mitotic activity (*p* = 0.006) and with severe nuclear atypia (*p* < 0.001). In these cases, a larger maximum diameter was associated with metastasis/recurrence (*p* = 0.028). Lymphovascular invasion was associated with an increased number of mitoses (*p* = 0.001), with the presence of necrosis (*p* = 0.020), an increased average of maximum diameter (*p* = 0.005), and an increased microvascular density (*p* = 0.034). The increase in the severity of nuclear atypia was associated with increased mitotic activity (*p* = 0.020), as well as with the presence of hemorrhage (*p* = 0.032). A statistically significant association was observed between the presence of arterial hypertension and the presence of metastases (*p* = 0.005).

**Figure 4:**
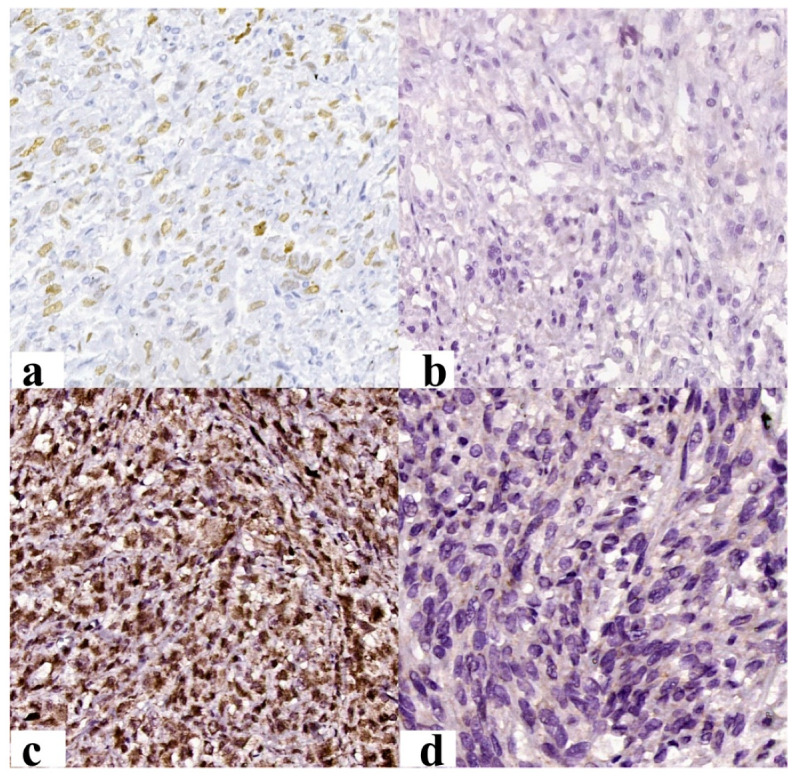
Immunohistochemical results: (**a**) Intense, diffuse positive reaction of p53 in more than 80% of the nuclei (Ob. 200×); (**b**) the negative reaction of p53 (Ob. 200×); (**c**) intense, diffuse positive nuclear reaction of RB1 (Ob. 200×); (**d**) the negative reaction of p53 (Ob. 200×).

Most cases (73.91%) presented a p53-negative reaction. In immunopositive cases, an average of positive nuclei of 35.71% for symplastic leiomyomas and 20% for leiomyosarcomas was observed (Figure 4a,b). Samples with an increased percentage of immunoreaction were associated with advanced age at diagnosis (*p* = 0.043).

The immunoreaction to Rb1 was positive in 56.52% of cases, with the average number of positive nuclei identified being 21.52% (Figure 4c,d). The diagnosis of mitotically active leiomyoma was associated with immunopositivity for Rb1, while that of leiomyoma with bizarre nuclei was associated with immunonegativity for Rb1 (*p* = 0.019) (Table 4). An increased nuclear percentage was associated with a high FIGO score (*p* = 0.003). The negative immunoreaction was associated with a faster onset of symptoms (*p* = 0.021). In benign cases, an increased nuclear percentage was associated with an inflammatory infiltrate rich in CD8-positive cells (*p* = 0.003) and with a low maximum diameter (*p* = 0.036).

**Figure 5:**
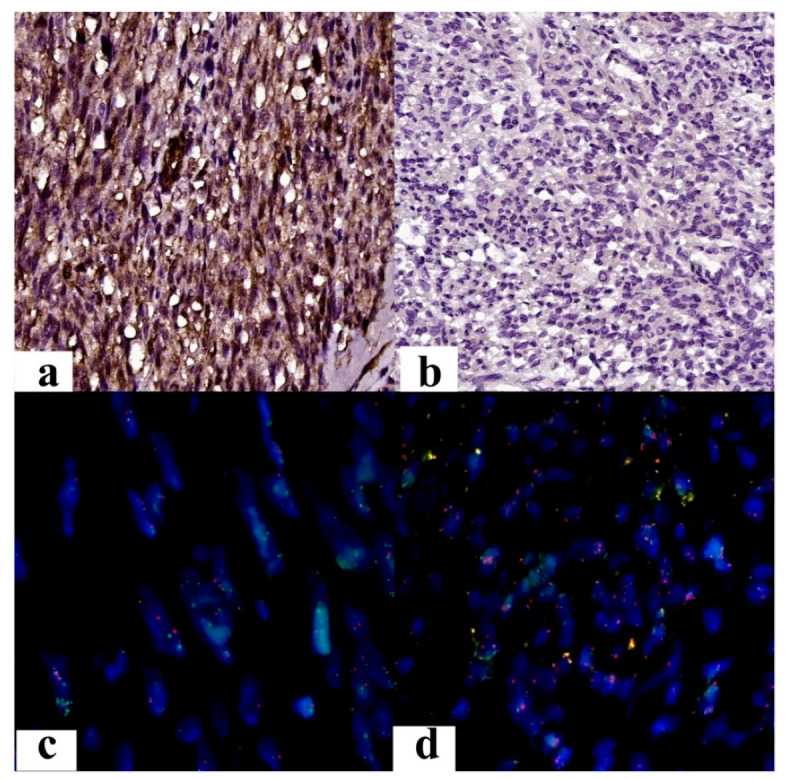
Immunohistochemical results of PTEN: (**a**) Intense, diffuse positive reaction (Ob. 200×). (**b**) The negative reaction of PTEN (Ob. 200×). Representative photomicrograph of FISH analysis of *PTEN* gene (orange signals) in a paraffine section, showing: (**c**) the normal status of the gene, with 2 green and 2 orange signals (Ob. 400×), and (**d**) deletion of *PTEN*, with 1 or no green signal and 2 orange signals (Ob. 400×).

Immunoreactivity to PTEN was identified in 34.78% of cases (Figure 5a,b), revealing a statistically significant difference between diagnosis and immunoreaction (*p* = 0.009). From a cytogenetic point of view, deletion of the *PTEN* gene was observed in 65.22% of cases (Figure 5c,d).

Also, the same diagnostic categories that presented severe atypia were associated with a negative PTEN immunoreaction (*p* = 0.034). Gene deletion was associated with a high FIGO (*p* = 0.002). The unaltered status of the gene was associated with the immunopositivity of the Rb1 marker (*p* = 0.029).

**Figure 6:**
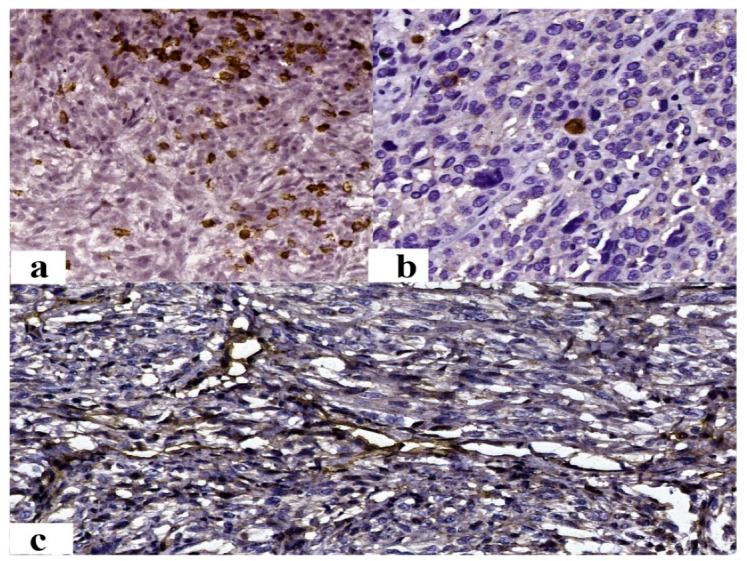
Immunohistochemical staining of: (**a**) CD8—intense, positive membranous reaction in CD8+ T cells (Ob. 200×); (**b**) PD-L1—intense, positive membranous reaction in tumor-immune lymphocytes (Ob. 200×); (**c**) CD105 (endoglin)—intense, positive membranous reaction in the endothelium (Ob. 200×).

The presence of intratumoral CD8+ inflammatory infiltrate was identified in 82.61% of cases, with an average of 16.55/mm^2^ positive cells (Figure 6a). A statistically significant difference was observed between the average value of CD8-positive inflammatory infiltrate and the tumor type. Most positive reactions were observed in the case of leiomyosarcoma, followed by symplastic leiomyoma and STUMP (*p* = 0.025) (Table 4). Also, an increased average was associated with an advanced stage at diagnosis (*p* = 0.038) and with the presence of tumor necrosis (*p* = 0.018). Also, it was associated with menopause (*p* = 0.009) and the presence of diabetes (*p* = 0.012). Moreover, the increased average of CD8+ T cells correlated with an increased percentage of Rb1+ nuclei (*p* = 0.003). In the case of malignant tumors and those with uncertain biological potential, an association was observed with the severity of atypia (*p* = 0.003) and the risk of metastasis (*p* = 0.009).

The PD-L1 immunoreaction at the level of the tumor inflammatory infiltrate was present in 82.61% of cases, showing an average of 1.17% (Figure 6b). In the cases of benign tumors included in the study, the absence of immunoreaction for PD-L1 was statistically significantly associated with the presence of dyslipidemia (*p* = 0.006), diabetes (*p* = 0.024), and hypertension (*p* = 0.040). On the other hand, the positive immunoreaction of PD-L1 was associated with thyroid dysfunction (*p* = 0.024). An increased average value of PD-L1 in the tumor inflammatory infiltrate was associated with a high FIGO score (*p* = 0.002) in malignant tumors, as well as an increased percentage of Rb1-positive nuclei (*p* = 0.047) in all entities. In cases of malignant tumors, an increased average value of PD-L1-positive T cells was associated with shorter survival (*p* = 0.043).

Regarding microvascular density, an average of 5.54 vessels/mm^2^ was observed, without a statistically significant distribution, depending on the diagnosis (*p* = 0.343) (Table 4) (Figure 6c).

## 4. Discussion

Uterine smooth muscle tumors are the most common mesenchymal tumors diagnosed in gynecological pathology and can cause differential diagnosis problems. Benign tumors, especially their variants, present a variety of morphological characteristics that cause problems in differential diagnosis with their malignant equivalent, leiomyosarcoma [7]. Immunohistochemical tests can help differentiate leiomyoma variants and STUMP from leiomyosarcoma, but these evaluations are not sufficiently specific for differential diagnosis, with additional investigations being necessary [8].

Mitotically active leiomyoma is diagnosed in the pre-menopausal period. Leiomyoma with bizarre nuclei occurs at an average age of 42.5–49.8, 10–15 years earlier than for leiomyosarcoma [9,10]. In the study conducted by Kefeli M et al., the authors included 30 cases of patients aged between 38 and 89 years, with an average of 49.76 years [11]. Another study that included 59 cases of symplastic leiomyoma reported an age range of 25–75 years, with an average of 45 years [12]. In our study, the average age at the time of diagnosis was 44.75 years (41–47) for mitotically active leiomyoma, with all patients being in the premenopausal period. Regarding atypical leiomyomas, the average age was 45 years (33–54), with the majority of patients being in the fertile period (71.43%).

The definition given by the WHO (2020) for STUMP describes smooth muscle tumors whose microscopic characteristics indicate potential for malignancy, but which do not meet the diagnostic criteria for either leiomyoma or leiomyosarcoma [8]. These represent 1.3% of all uterine malignant neoplasms [13]. They are diagnosed more frequently in premenopausal women, with an average age of 44 years [14]. The incidence of these tumors is not very well documented, varying by around 0.01% in patients treated surgically for the presumptive diagnosis of uterine leiomyoma [15]. In the current research, we observed that the age at diagnosis of patients with this tumor varied between 43 and 48 years, with an average of 45. All patients were in their fertile period at the time of diagnosis.

Leiomyosarcoma is the most common uterine sarcoma. It contributes to approximately 70% of these tumors. It can be associated with obesity and diabetes [16]. Studies in the specialized literature report incidence rates between 0.35 and 0.8/100.000 women [16,17,18]. This can increase to 17/100.000 women in the case of tamoxifen treatment for a longer period of 5 years [16]. The prevalence of uterine sarcomas is 3–7/100.000 [19]. Most patients do not present predisposing factors, but the potential risk factors are: prior radiation therapy to the pelvis (10–25%), long-term tamoxifen (1–2%), or certain inherited genetic syndromes, and postmenopausal period [20]. In the current study, it was observed that the patients with this diagnosis were aged between 43 and 75 years, with an average of 54.56. The main comorbidities were hypertension (66.67%) and dyslipidemia (55.56%), followed by diabetes and altered thyroid function.

The symptoms determined by STUMP are not specific, and are similar to those of leiomyomas: metrorrhagia, pelvic mass, and pelvic pain, but also secondary symptoms of compression phenomena or anemia [13]. Leiomyosarcomas manifest at the time of diagnosis as metrorrhagia (56%), a palpable pelvic mass (54%), abdominal pain (22%), abnormal vaginal secretions, pollakiuria, constipation, and abdominal distension [21,22]. Less often, it is diagnosed secondary to hemoperitoneum, with symptoms generated by extra-uterine extension or distant metastases [22]. For all tumors included in this study, the most common clinical manifestations highlighted were metrorrhagia and pelvic pain. This aspect underlines the non-specificity of the manifestations and, at the same time, the importance of histopathological examinations and those complementary to them.

Hence, uterine smooth muscle tumors form a heterogeneous group of pathological entities, which can pose problems in establishing the final diagnosis. From a molecular point of view, leiomyomas are considered clonal neoplasms different from leiomyosarcomas [23].

From a pathogenic point of view, the main pathways involved are those of the *p53*, *RB1*, and *PTEN* genes [24]. Thus, a loss of *p53* function is involved in affecting the inhibition of the metastasis pathway. This alteration seems to contribute to weakening of intercellular junctions and the destruction of epithelial integrity, thus favoring the dissemination of cells that make up solid tumors [25]. The *RB1* gene is located in the 13q region. The role of the *RB1* gene is to prevent progression from the G1 phase to the S phase of the cell cycle [26]. Regulatory aberration in this region is known to induce cells to divide indefinitely [27]. Genetic deficiency in chromosome 10q means the inactivation of tumor suppressor gene *PTEN*, leading to activation of the PI3K/AKT pathway and the downstream mTOR pathway [27]. This pathway has a role in numerous neoplastic processes, such as cell growth and resistance to chemotherapy [28].

There are multiple studies according to which the frequency of *TP53* mutations is 0% in leiomyomas, 6–29% in STUMP, and 24–30% in leiomyosarcomas. Also, the reported percentages for *PTEN* gene mutations are 5% in the case of leiomyomas, 33% in STUMP cases, and 42–58% in leiomyosarcomas [27]. In the study carried out by Astolfi et al., which included 216 cases of leiomyosarcoma, they observed that the frequency of *RB1* gene mutations was 48%. In addition, the authors observed that *PTEN* gene mutations are more frequent in cases of metastasis than in primary tumors. In their study, *TP53* and *RB1* mutations appeared together, with the latter having favorable prognostic significance in cases with *TP53* mutations present [29].

Zhang et al. studied the frequency of *TP53* and *PTEN* gene mutations, highlighting similar frequencies between cases of leiomyosarcoma and those of STUMP and atypical leiomyoma. They also observed that leiomyomas with bizarre nuclei had several molecular alterations similar to leiomyosarcomas, suggesting that this type of leiomyoma could be the precursor of malignant tumors [27]. From a molecular point of view, leiomyomas are considered to represent separate clonal neoplasms, which occur independently of leiomyosarcoma. The overlap in genomic changes in atypical leiomyoma and leiomyosarcoma suggests that they develop through similar pathogenic pathways, with the former being a precursor to leiomyosarcoma after acquiring additional alterations. The difference between the ages at which the two entities appear suggests a potential time-dependent relationship [23]. The results of our study are consistent with this idea. The values of the analyzed parameters are similar between leiomyosarcoma, STUMP, and atypical leiomyoma. For example, it was observed that the frequency of *TP53* and *PTEN* gene mutations had more appropriate values between leiomyosarcomas, STUMP, and atypical leiomyoma compared to mitotically active leiomyoma. Also, the age difference between leiomyoma with bizarre nuclei and leiomyosarcoma was, on average, 9.56 years, varying between 10 and 21 years. These data are in agreement with the literature, and support the idea that atypical leiomyoma can be a precursor to leiomyosarcoma, with the relationship between the two being time-dependent.

There are data suggesting that *PTEN* has a role as an initiator of anti-tumor immunity. In patients diagnosed with tumors with *PTEN* deletion, increased values of inflammatory pro-oncogene cytokines and immunosuppressive cells, as well as low levels of NK cells, T helper, and cytotoxic lymphocytes, were observed [30].

The tumor microclimate is a specialized environment formed by host components and structured by tumor cells to maintain tumor development and possible dissemination [3]. It consists of blood and lymphatic vessels, stroma, and immune cells. They are present in the affected tissue or recruited from its periphery [31]. The dynamic interaction between neoplastic cells and those components of the tumor microclimate has a major role in the emergence of heterogeneity, clonal progression, and the acquisition of resistance to chemotherapy [3]. During this interaction, both tumor and stromal cells can change their immunophenotype with tumor progression [5].

From the cytokine superfamily, some of its members have CD105 as their receptor. it has increased endothelial expression during active angiogenesis, especially at the angiogenic edges [32,33,34]. By evaluating the microvascularization using immunohistochemical tests, an association was observed between its high density and the poor prognosis of some solid tumors [35,36]. Ollauri-Ibanez C et al. demonstrated that the constitutive overexpression of endoglin affects the maturation and stabilization of blood vessels. The tumoral consequence of this change is expressed by the extravasation of red blood cells in the tumor stroma, but also the intravasation of malignant cells, without influencing the size of the tumor. Thus, by administering targeted treatments for endoglin, the alteration of the vascular spaces can be reduced and the sensitivity to chemotherapy can be increased [37]. Some studies have highlighted the important role of CD105 in resistance to treatment. For example, its expression is associated with a reduced degree of differentiation, advanced stages of the disease, resistance to therapy, and high recurrence rates in ovarian cancer [38]. In the case of patients who received oncological treatment, it was found that the number of months until death was higher in cases with higher average values of CD105-positive intratumoral capillaries. Through this observation, the current study supports and emphasizes the importance of evaluating the density of tumor vascularization, for identifying the optimal treatment path or for increasing the sensitivity to other chemotherapeutics administered.

Also, CD105 favors the epithelial–mesenchymal transition [38]. In the case of sarcomas, this phenomenon is mostly unknown and paradoxical, because their origin is mesenchymal. Sannino et al. suggested that sarcomatous cells can be in a metastatic state and that their differentiation towards an epithelial phenotype or a predominantly mesenchymal phenotype depends on the cellular context. This characteristic was correlated with an aggressive phenotype [39].

Malignant cells secrete antigens that determine the activation of CD8+ lymphocytes that will migrate into the tumor microclimate [40]. The tumoral lymphocytic infiltrate consists mostly of CD4- and CD8-positive lymphocytes. The latter produce large amounts of pro-inflammatory cytokines, having a highly cytotoxic effect on tumor cells. PD-1 is a receptor expressed by T lymphocytes, and its ligand (PD-L1) is overexpressed by malignant cells to decrease the host’s immune response [41].

PD-L1 expression is regulated by extrinsic and intrinsic signals, such as the loss of *PTEN* gene function or the activation of the pathogenic PI3K/Akt pathway, both found in the pathogenesis of uterine leiomyosarcomas [42,43]. Ben-Ami, E., et al. observed, in their study, that the reduced number of CD4- and CD8-positive correlated with low values of PD-L1 [44]. In the case of our study, the correlation of PD-L1 values with those of CD8 or with the deletion of the *PTEN* gene was not highlighted. Instead, a correlation of the IC values of PD-L1-positive lymphocytes with the increased percentage of Rb1+ nuclei was observed, in addition to reduced survival.

Shanes ED et al. studied PD-L1 expression in 49 uterine smooth muscle tumors: 11 typical leiomyomas, 7 atypical leiomyomas, 8 STUMP, and 23 leiomyosarcomas. They observed positive tumoral PD-L1 expression in 70% of leiomyosarcomas and 14% of leiomyomas with bizarre nuclei. The reaction was negative in cases of typical leiomyoma and STUMP [45]. In the case of our study, a positive reaction of PD-L1 was observed in all evaluated cases: 88.89% of leiomyosarcomas, 66.67% of STUMP, 85.71% of leiomyomas with bizarre nuclei, and 75% of mitotically active leiomyomas. Regarding its presence at the level of immune cells associated with the tumor, the reaction was present in 78% of leiomyosarcomas, 25% of STUMP, and 9% of typical leiomyomas, without being observed in atypical leiomyomas. The results they obtained suggest the possibility of administering targeted immunotherapy to patients diagnosed with leiomyosarcoma [45].

Type 2 diabetes is characterized by defective glucose metabolism secondary to insulin resistance. This condition is associated with a chronic systemic inflammatory state, of reduced intensity [46]. Malignant cells show alterations in the metabolism of glucose and amino acids in response to an accelerated rate of cell proliferation [47]. It has been observed that diabetes is associated with increased mortality from any cancer in 7% of men and 11% of women [48]. However, there are also such pathologies for which the association with diabetes is null, such as cervix, esophagus, and bladder leukemia [48]. The neoplastic process can be supported by the presence of diabetes through hyperinsulinemia and hyperglycemia, for the nutrition of cancer cells through aerobic glycolysis. In clinical and experimental models, it has been observed that PD-L1 expression is associated with intense carbohydrate metabolism [49].

In the study carried out by Febres-Aldana CA et al., it was observed that in diabetic patients diagnosed with non-small-cell lung carcinoma, the peritumoral inflammatory infiltrate was more pronounced, and PD-L1 positivity was higher compared to those in non-diabetic patients [49]. In the tumor microclimate, T lymphocytes fail to fulfill their anti-tumor role secondary to their chronic activation, inadequate stimulation, and the absence of antigen recognition. Also, there is a reduction in glycolysis and cytokine synthesis, and a decrease in the activity of the mTOR pathway following the metabolic competition between T lymphocytes from the tumor infiltrate and malignant cells. The experiments showed a reduction in the basal respiration/glycolysis ratio, as well as impairment of the multiple roles of CD8+ PD-1+ T lymphocytes in the tumor. This suggests that the development and advancement of tumors are supported by alteration of the immune function-metabolism link of CD8+ PD-1+ T lymphocytes [47]. In our study, a significant association was observed between the average value of CD8+ T lymphocytes and the presence of diabetes (*p* = 0.012).

The differential diagnosis between symplastic leiomyoma, mitotically active leiomyoma, STUMP, and leiomyosarcoma is based on three criteria: mitotic activity, nuclear atypia, and tumor necrosis. There are three types of necrosis observed in uterine muscle tumors: in the case of submucous localization, ulceration and underlying necrosis are identified, infarct-type necrosis is evident in both benign and malignant tumors, and tumor cell necrosis is visualized only in leiomyosarcoma [6].

Mitotically active leiomyoma has, as its morphological characteristic that can cause differential diagnosis problems, the presence of increased mitotic activity, especially when more than five mitoses/10 HPFs are identified. Tumor necrosis is not usually identified. Unlike leiomyosarcomas, they almost always appear in women during the reproductive period, being associated with the secretory phase of the menstrual cycle, pregnancy, or the exogenous administration of hormones [6]. In our case, all patients with this diagnosis were premenopausal, with 75% of them having had previous pregnancies.

Also called symplastic leiomyoma, leiomyoma with bizarre nuclei poses problems of differential diagnosis with leiomyosarcoma, because it is characterized by the presence of uni- or multinucleated, large, atypical cells, with a diffuse distribution within the tumor. They can also present hypercellular areas and prominent nucleoli, but also karyorrhectic nuclei and granular chromatin, mimicking atypical mitoses. Their mitotic activity is focally increased to up to eight mitoses/10 HPFs. Tumor necrosis is usually absent [6].

The criteria that allow a uterine muscle tumor to be classified as STUMP are varied: diffuse nuclear atypia, with two–four mitoses/mm^2^ and the absence of necrosis; tumor necrosis present, without other worrisome microscopic features; more than six mitoses/mm^2^ with the absence of tumor necrosis and cytological atypia; and diffuse nuclear atypia and uncertain mitotic number secondary to accentuated karyorrhexis phenomena [1]. Considering the diversity of criteria for inclusion in this diagnostic category, it is essential to perform a correct differential diagnosis by using all the necessary ancillary examinations.

In cases of mitotically active leiomyoma and leiomyoma with bizarre nuclei included in this study, areas of hemorrhage and necrosis were not identified, and the average values of the number of mitoses were 11.85 and 2.43, respectively. For the diagnosis of STUMP, necrosis and hemorrhage were observed in one case each, with the value of mitoses varying between 5 and 12 mitoses/10 HPFs. However, necrosis prevailed in malignant tumors (88.89%), and hemorrhage was rarely evident (22.22%). The nuclear atypia was severe, and the average mitotic activity was 15.56 (5–35 mitoses/10 HPFs). These similar data support the importance of differential diagnosis, because therapeutic options, evolution, and prognosis are extremely varied.

Currently, the therapeutic options in cases of symptomatic leiomyoma are represented by expectation, medication, or surgical intervention [50]. The main treatment in the case of leiomyosarcomas is surgery (total hysterectomy, in cases with disease limited to the uterus). The role of adjuvant radiotherapy in such cases is controversial. Due to the absence of obvious benefits, this therapeutic modality is not recommended in cases with optimal surgical resection. For patients with advanced disease, with incomplete resection or metastasis, adjuvant radiotherapy is more useful for palliative purposes. Although chemotherapy administered post-operatively has a poorly defined role in the early stages of the disease, it is used because of the high risk of systemic recurrence. In addition, chemotherapy is a viable option for patients with metastatic disease, in cases where the functioning of the organs allows for the use of these substances. Immunotherapy based on anti-PD-1 antibodies is under study [20].

According to the NCCN (National Comprehensive Cancer Network) guide, monitoring the patient’s progress should involve a physical examination every 3–4 months in the first 2–3 years, and then, every 6–12 months. Imaging investigations should include thoracic–abdominal and pelvic CT at 3–6 months in the first 3 years, and at 6–12 months in the following 2 years. These can be performed for another 5 years, once or twice a year, depending on the microscopic aspects and the stage of the disease at diagnosis [20].

The recurrence rates of STUMP cases range from 7% to 27%. These tumors can relapse later in the evolution of patients, after an average period of 51 months [13]. Other authors claim that the recurrence rate is, depending on the tumor subtype, between 7.3% and 26%, with a total recurrence rate of 11% [51]. STUMP is characterized by slow, silent evolution, with deaths caused by this tumor being rare. Nevertheless, cases with rapid, unfavorable clinical courses following the appearance of metastases have also been described [52,53,54]. Rizzo A et al. observed, in their study, that survival after the initial diagnosis of STUMP was, on average, 101 months [55].

The recurrence rates for leiomyosarcoma cases range from 45% to 75%, with the most common sites of metastasis being the lungs. A review of the literature showed that recurrence rates range from 45% to 73% [56]. Considering leiomyosarcomas, survival rates after a period of 5 years vary from 25% to 76%, dropping to 10–15% if distant metastases are present at the time of initial diagnosis [17].

In the current study, only the leiomyosarcoma cases presented metastases with a percentage of 77.78%. The survival of these patients had an average of 25.67 months, with the relative risk of these cases by comparison being 5.66 (*p* < 0.001, CI95 2.02–15.82). No deaths or recurrences were identified in cases of patients diagnosed with STUMP.

The strong points of this study are represented by the small number of recent studies that include all these entities, but also the fact that we found, in the literature, few studies that associate pathogenic pathways with the tumor microclimate, and then, compare them between different categories of uterine smooth muscle tumor. Also, our study correlates the data obtained with the patients’ comorbidities, highlighting their importance for women’s public health. Thus, more studies are required that take into account these parameters, both in the differentiation of tumor entities and in the evaluation of the most optimal treatment methods, along with surgical ones. The limitations of our research are represented by its retrospective nature and the small number of identified cases.

## 5. Conclusions

Despite the fact that uterine myocyte tumors have been intensively studied in recent years, so far, no consensus has been reached regarding the involvement of the tumor microclimate in evolution and prognosis, but also the impact of genetic mutations on the tumor environment. Moreover, the idea that symplastic leiomyoma could be a precursor to leiomyosarcoma requires the performance of prospective studies in specialized centers. Clarifying this idea could lead to the prevention of cases of uterine leiomyosarcoma and to significant improvements in patients’ quality of life.

The current study points out the importance of evaluating the evolutionary stages of pathogenesis and tumor microvascular density in the treatment of these pathological entities. The obtained results support the recent specialized literature, but besides this, they provide information regarding the apparent harmlessness of benign lesions, as well as the deceptive appearance of lesions with uncertain biological potential. Thus, the complete and correct evaluation of all cases by highlighting differential diagnoses is necessary because the histogenetic aspects (the tumor microclimate, tumor microvascularization, pleomorphism, and cytonuclear atypia), combined with the clinical elements, have, as their final goal, effective guidance of the therapeutic management, which has an impact on improving the quality of women’s lives.

## Figures and Tables

**Table 1:** Interpretation and results of immunohistochemical tests.

Antibody (Clone)	Reaction	Quantification
p53 (SP5)	Nuclear	Qualitative: present/absent reaction Quantitative: overexpression ≥80% nuclei; null 0%; wild-type 0–80% (patchy distribution)
RB1 (1F8)	Nuclear	Qualitative: present/absent reaction Quantitative: 10 HPF fields (40×) reported per 1000 nuclei evaluated
PTEN (6H2.1)	Nuclear	Qualitative: present/absent reaction Quantitative: 10 HPF fields (40×) reported per 1000 nuclei evaluated
CD8 (SP16)	Membranous	Qualitative: present/absent reaction Quantitative: mean number of T cells/1 mm^2^
PD-L1 (CAL10)	Membranous	Qualitative: present/absent reaction Quantitative: the area occupied by PD-L1-positive lymphocytes relative to the entire tumoral and peritumoral area
CD105 (3A9)	Membranous	Quantitative: mean number of intratumoral CD105-positive capillaries/1 mm^2^

**Table 2:** Clinical description of evaluated cases.

	Leiomyosarcoma	STUMP	Symplastic Leiomyoma	Mitotically Active Leiomyoma	*p* Value
Age (mean)	43–75 (54.56)	43–48 (45)	33–54 (45)	41–47 (44.75)	0.057
Symptoms (*n*/N)					
Metrorrhagia	78% (7/9)	67% (2/3)	43% (3/7)	75% (3/4)	0.572
Pelvic pains	67% (6/9)	0% (0/3)	86% (6/7)	50% (2/4)	0.090
Hypermenorrhea	0% (0/9)	67% (2/3)	0% (0/7)	0% (0/4)	0.012
Debut (*n*/N)					
<1 week	89% (8/9)	0% (0/3)	43% (3/7)	0% (0/4)	0.001
1 week–1 month	11% (1/9)	100% (3/3)	14% (1/7)	25% (1/4)	
>1 month	0% (0/9)	0% (0/3)	43% (3/7)	75% (3/4)	
Hormonal status (*n*/N)					
Premenopause	22% (2/9)	100% (3/3)	71% (5/7)	100% (4/4)	0.016
Menopause	78% (7/9)	0% (0/3)	29% (2/7)	0% (0/4)	
Comorbidities (*n*/N)					
Hypertension	67% (6/9)	0% (0/3)	29% (2/7)	50% (2/4)	0.229
Dyslipidemia	56% (5/9)	67% (2/3)	29% (2/7)	25% (1/4)	0.554
Diabetes	22% (2/9)	0% (0/3)	0% (0/7)	25% (1/4)	0.502
Non-euthyroidism	22% (2/9)	0% (0/3)	0% (0/7)	25% (1/4)	0.834
Parity (*n*/N)					
Parous	44% (4/9)	67% (2/3)	71% (5/7)	75% (3/4)	0.726
Nulliparous	56% (5/9)	33% (1/3)	29% (2/7)	25% (1/4)	

**Table 3:** Macroscopical characteristics and their correlations.

	Leiomyosarcoma	STUMP	Symplastic Leiomyoma	Mitotically Active Leiomyoma	*p* Value
Surgical treatment (*n*/N)					
Total hysterectomy	78% (7/9)	100% (3/3)	71% (5/7)	50% (2/4)	0.367
Subtotal hysterectomy	11% (1/9)	0% (0/3)	0% (0/7)	0% (0/4)	
Myomectomy	0% (0/9)	0% (0/3)	29% (2/7)	50% (2/4)	
Hysterectomy with adnexectomy	11% (1/9)	0% (0/3)	0% (0/7)	0% (0/4)	
Location (*n*/N)					
Intramural	100% (9/9)	67% (2/3)	43% (3/7)	50% (2/4)	0.014
Subserous	0% (0/9)	0% (0/3)	57% (4/7)	25% (1/4)	
Submucosal	0% (0/9)	33% (1/3)	0% (0/7)	25% (1/4)	
Maximum diameter (cm)	11.5	5.33	6.35	7.75	0.091
	5.5–28	3–7	1.5–10	5–14	

**Table 4:** Results and associations of microscopical and immunogenetic aspects.

	Leiomyosarcoma	STUMP	Symplastic Leiomyoma	Mitotically Active Leiomyoma	*p* Value
Number of mitoses (mean)	5–35 (15.56)	5–12 (8.67)	1–4 (2.43)	8–15 (11.25)	0.002
Presence of necrosis (*n*/N)	89% (8/9)	33% (1/3)	0% (0/7)	0% (0/4)	<0.001
Presence of hemorrhage (*n*/N)	22% (2/9)	33% (1/3)	0% (0/7)	0% (0/4)	0.421
p53 (*n*/N)					
Overexpressed	22% (2/9)	0% (0/3)	43% (3/7)	0% (0/4)	0.544
Null-type	67% (6/9)	100% (3/3)	57% (4/7)	100% (4/4)	
Wild-type	11% (1/9)	0% (0/3)	0% (0/7)	0% (0/4)	
PTEN+ (*n*/N)	11% (1/9)	0% (0/3)	43% (3/7)	100% (4/4)	0.009
*PTEN* deletion (*n*/N)	89% (8/9)	100% (3/3)	57% (4/7)	0% (0/4)	0.009
Rb1+ (*n*/N)	44% (4/9)	100% (3/3)	29% (2/7)	100% (4/4)	0.049
Average	24.44	36.67	16.43	12.5	0.545
CD8+	3–87.89 (30.25)	0–10.52 (6.84)	0–23.15 (9.60)	0–9.47 (5.21)	0.025
PD-L1+	0–3%	0–2%	0–3%	0–2%	0.799
Average	1%	1%	1%	1%	
Microvascular density (mean)	2.17–11.26 (6.44)	5.89–8.42 (6.93)	0.25–9.04 (3.85)	0.89–10.51 (5.42)	0.343

## Data Availability

Not applicable.
